# Can Physical Activity and Healthy Diet Help Long-Term Cancer Survivors Manage Their Fear of Recurrence?

**DOI:** 10.3389/fpsyg.2021.647432

**Published:** 2021-06-09

**Authors:** Caroline Séguin Leclair, Sophie Lebel, J. Lee Westmaas

**Affiliations:** ^1^School of Psychology, University of Ottawa, Ottawa, ON, Canada; ^2^Behavioral Research Center, American Cancer Society, Atlanta, GA, United States

**Keywords:** cancer survivors, common-sense model, diet, fear of cancer recurrence, self-efficacy, physical activity

## Abstract

**Objective**: Fear of cancer recurrence (FCR) adversely affects quality of life, but health behaviors such as physical activity (PA) and fruit and vegetable intake (FVI) may help alleviate FCR for some survivors. This cross-sectional study tested the common-sense model (CSM) of FCR by investigating associations between constructs from the CSM (perceived illness consequences, control over health, and timeline), and survivors’ health behaviors, health self-efficacy, and FCR.

**Methods**: Using wave 3 data from the American Cancer Society Longitudinal Study of Cancer Survivorship-I, path analyses were conducted among mixed-cancer participants (*N* = 2,337) who were on average 8.8 mean years post-diagnosis.

**Results**: A final good fitting model [*χ*^2^ (5, *N* = 2,337) = 38.12, *p* < 0.001; SRMR = 0.02; CFI = 0.99; RMSEA = 0.05] indicated that perceiving fewer illness consequences, and greater control over one’s health, were directly associated with higher PA (*β* = 0.15 and −0.24, *p* < 0.01, respectively) and higher health self-efficacy (*β* = 0.24, −0.38, *p* < 0.01, respectively). Timeline (i.e., perceiving cancer as chronic) was directly associated with lower health self-efficacy (*β* = −0.15, *p* < 0.01) and higher FCR (*β* = 0.51, *p* < 0.01). Both greater PA and FVI were directly associated with higher health self-efficacy (*β* = 0.10 and 0.11, *p* < 0.01, respectively) which in turn showed a direct association with lower FCR (*β* = −0.15, *p* < 0.01).

**Conclusion**: Increasing survivors’ sense of control over health, decreasing perceived chronicity of the illness, and mitigating its consequences may increase their health behaviors and health self-efficacy, which in turn could decrease their FCR. Longitudinal and experimental studies are needed to confirm these findings.

## Introduction

The population of cancer survivors is growing in North America (American Cancer Society, 2016). After cancer treatment, cancer survivors are left facing several psychosocial challenges, including fear of cancer recurrence (FCR; Simard et al., 2013; Simonelli et al., 2017). FCR is defined as the fear, worry, or concern relating to the possibility that cancer will come back or progress (Lebel et al., 2016). Thus far, FCR research has mainly focused on identifying detrimental coping responses to FCR (i.e., reassurance seeking, body checking, and avoidance), which contribute to the maintenance of cancer survivors’ distress. Helpful coping responses to manage FCR, however, remain understudied (Simard et al., 2013).

With the growing body of evidence demonstrating that lifestyle changes have a countering effect on cancer progression/recurrence and promote healthy survivorship (Pekmezi and Demark-Wahnefried, 2011), cancer survivors are encouraged by health care practitioners to engage in health behaviors (i.e., physical activity and healthy diet). Specifically, the American Cancer Society recommends that cancer survivors engage in 150 min of moderate physical activity or 75 min of vigorous physical activity weekly and the intake of at least five portions (5-a-day) of fruits and vegetables each day (Kushi et al., 2012).

While the role of health behaviors in reducing the risk of cancer recurrence in survivors is well-established, little is known of their impact on FCR. Specifically, can engaging in health behaviors help survivors manage their FCR? Investigating relationships between FCR and health behaviors is a first, necessary step before further testing the hypothesized role of health behaviors as a positive coping strategy in longitudinal or experimental studies. Leventhal’s common-sense model (CSM) of self-regulation and Bandura’s self-efficacy theory were used as theoretical frameworks to examine these relationships.

### Conceptualizing FCR Using the Common-Sense Model

The CSM is the most comprehensive and evidenced-based theoretical approach applied to FCR (Fardell et al., 2016). Originally developed to encompass the cognitive, behavioral, and emotional responses to various illnesses (Leventhal et al., 1992), Lee-Jones et al. (1997) applied the CSM components to the context of cancer in their FCR theoretical formulation. The CSM components have since been empirically validated in cancer survivors (Fardell et al., 2016; Simonelli et al., 2017). According to this theoretical formulation, when an *illness threat* (triggers, i.e., aches and pains) is perceived, it activates the cancer survivor’s *illness representation* informing the selection of *coping* response, which will ultimately influence the illness and emotional *outcomes*, including FCR (Leventhal et al., 1992; Lee-Jones et al., 1997).

The illness representation is comprised of five illness attributes: *illness identity* – refers to the illness label (cancer) and related symptoms (e.g., fatigue); *consequences* – refers to the perceived impact of cancer on an individual’s life, including social, psychological, and physical consequences (e.g., impact on family); *control* – refers to the perceived level of control over cancer or curability by oneself or others (e.g., incurable, recurrence preventable); *timeline* – refers to the perceived time frame of cancer growth, illness course, and recovery (e.g., acute, chronic, or cyclical); and *causes* – refers to the perceived cause of cancer (e.g., stress, unhealthy lifestyle, or family history; Leventhal et al., 1992).

These attributes will inform the coping response chosen by the patient to manage emotional (typically with emotion focused coping) and/or illness outcomes (typically with problem-focused coping; Hagger et al., 2017). In samples of chronically ill patients, illness identity, consequences, and timeline are often correlated with emotion-focused coping, while control is more related to problem-focused coping (Richardson et al., 2016; Hagger et al., 2017). Health behaviors are generally conceptualized as problem-focused coping to manage illness outcomes (Richardson et al., 2016; Hagger et al., 2017); however, they have been shown to help manage emotional outcomes such as depression and quality of life (Aguiñaga et al., 2018). Therefore, from a theoretical standpoint, it is possible that health behaviors can help manage FCR, an emotional outcome.

### Conceptualizing Health Behaviors as a Coping Strategy

Based on the CSM, if health behaviors are a coping response to FCR, cancer survivors with an illness representation that is indicative of a more severe illness (e.g., those who perceive cancer to be chronic or its consequences to be more significant) are expected to engage in more physical activity (PA) and fruit and vegetable intake (FVI). Subsequently, if these coping strategies are appraised as effective, FCR should be reduced. The few studies using the CSM framework to test the relationship between illness representation, PA and FVI, have yielded mixed results (Mullens et al., 2004; Costanzo et al., 2011; Burris et al., 2012; McGinty et al., 2012; Green et al., 2014), suggesting the need for further investigation.

Additionally, previous studies of FCR and health behaviors using the CSM were restricted to one or two disease sites and confined to early survivorship (i.e., 2 years post active treatment; Stanton et al., 2015) limiting their generalizability. Additional studies are required to clarify the contradictory findings using a large sample of survivors with a range of cancer diagnoses.

### Adding Self-Efficacy

In the context of cancer survivorship, self-efficacy is defined as the perceived confidence in handling problems related to one’s health (Bandura, 1997). In one study, the inclusion of self-efficacy improved the fit of the CSM in predicting personal control over illness in older adults with 10 different chronic diseases (Schüz et al., 2012). Additionally, several studies have found that self-efficacy played a mediating role in predicting FCR. For example, improvements in self-efficacy were found to mediate the effects of a brief communication intervention on FCR (Shields et al., 2010), between FCR vulnerability factors such as trait anxiety and cancer reminders and FCR in women with breast cancer (Ziner et al., 2012), and between physical symptoms and FCR in men with prostate cancer (Torbit et al., 2015). In the present study, health self-efficacy was conceptualized as the appraisal of the coping response (i.e., the health behaviors) and was expected to mediate the relationship between the coping and the emotional outcome, FCR.

### Study Objectives

**Figure 1:**
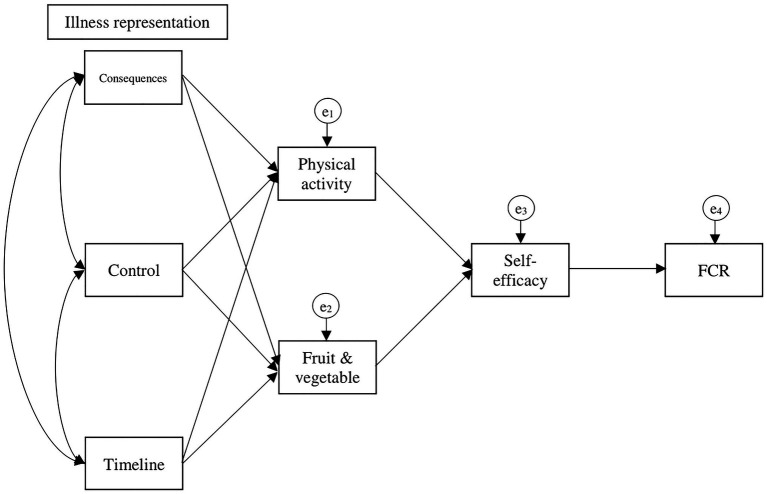
Hypothesized model based on the theoretical FCR common-sense model (Lee-Jones et al., 1997) and self-efficacy theory (Bandura, 1997).

This cross-sectional study aimed to explore the relationships between constructs from the CSM and self-efficacy theory, and health behaviors (PA and FVI) and FCR in a population-based sample of survivors of 10 cancers [tobacco use was not included in the current analyses as its relationship with FCR was the focus of a separate study by Westmaas et al. (2019)]. We used data from wave 3 of the American Cancer Society’s Study of Cancer Survivors-I (SCS-I) because only the wave 3 survey included assessment of three constructs from the CSM (described below) in addition to health behaviors, self-efficacy, and FCR. The following relationships were expected (see Figure 1):

1. Illness representation → health behaviors: cancer survivors who reported (a) more illness consequences, (b) more control, and (c) viewed cancer as chronic were expected to report more health behaviors. (2) Health behaviors → self-efficacy: survivors who endorsed more health behaviors were expected to report greater self-efficacy. (3) Self-efficacy → FCR: survivors who reported greater self-efficacy were expected to display lower FCR. Similar trends were hypothesized for both health behaviors, PA and FVI (Mullens et al., 2004; Costanzo et al., 2011; Burris et al., 2012; Green et al., 2014).

## Materials and Methods

### Procedure

The current cross-sectional study is part of a larger longitudinal study examining FCR and health behaviors (Séguin Leclair et al., 2019) using data from the American Cancer Society’s SCS-I, a national prospective longitudinal study of American cancer survivors with data collected in three waves beginning in 2000, T1, *M* = 1.3 years (*SD* = 0.32), T2, *M* = 2.2 years (*SD* = 0.34), and T3, *M* = 8.8 years (*SD* = 0.63) post cancer diagnosis. Participant eligibility criteria were the following: diagnosed with one of the 10 most highly incident cancers [prostate, breast, lung, colorectal, bladder, non-Hodgkin lymphoma (NHL), skin melanoma, kidney, ovarian, and uterine], over 18 years old at diagnosis, residing in one of the target states at the time of diagnosis, and diagnosed with a local, regional, or distant SEER Summary Stage cancer. Survivors were ineligible for the study if they were unable to complete the survey due to mental incompetence, unable to communicate in English or Spanish, or had terminal illness (Smith et al., 2007). The studies were approved by the Institutional Review Board of Emory University (Atlanta, GA, United States), for each state, including the Connecticut Department of Public Health Human Investigation Committee, and the University of Ottawa Research and Ethics Board (Ottawa, Ontario). Additional details on recruitment and methodology are available elsewhere (Smith et al., 2007).

### Measures

#### Socio-Demographic and Medical Characteristics

The following socio-demographic and medical variables were examined and controlled for: age at diagnosis, sex, ethnicity, education, cancer site, and cancer stage based on their known relationship with FCR (Séguin Leclair et al., 2019). Relationship status, family income, and occupation were included for sample description purposes only.

### Illness Representation Attributes

#### Illness Consequences

Illness consequences were measured using the Medical Outcomes Study Short Form – Physical Health subscale (Ware et al., 1996). Using a 5-point Likert scale, respondents indicated their perception of physical functioning, impact of health on various roles, bodily pain, and general health. For example, “*During the past 4 weeks, how much of the time did you accomplish less than you would like as a result of your physical health?*” This measure has good test–retest reliability after 2 weeks (*r* = 0.86) and construct validity (*r* = 0.91) with the original Medical Outcomes Study Form. Final scores ranging from 0 to 100 were obtained by computing items scores and comparing them to age-specific reference groups. Higher scores indicated less illness consequences.

#### Control

The 9-item Perceived Health Competence Scale (Smith et al., 1995; Arora et al., 2002) was used to determine respondents’ impression of their ability to control their health. Items were rated on 5-point Likert scales ranging from strongly disagree to strongly agree (Cronbach’s *α* = 0.87). For example, “*No matter how hard I try, my health just does not turn out the way I would like*.” Total scores were computed, with higher scores indicating less control over health.

#### Timeline

The perceived time frame of the cancer (i.e., acute vs. chronic) was assessed using the susceptibility subscale of the Revised Health Belief Model Scale (Champion, 1999). These three items used a 5-point Likert scale ranging from *strongly agree* to *strongly disagree*; respondents indicated their perceived susceptibility of getting a cancer recurrence. For example, “*It is likely that I will get cancer again*.” It showed good internal consistency (*α* = 0.87), good test–retest reliability after 6 weeks (*r* = 0.62), and good construct validity (*r* = 0.87–0.91) with the original Susceptibility subscale of the Health Belief Model Scale (Champion, 1999). Total scores were computed, with higher scores indicating higher perceived chronicity of cancer.

### Health Behaviors

#### Physical Activity

The Leisure-Time Exercise Questionnaire (LTEQ) was used in this study to assess PA. Respondents indicated the number of minutes they spent doing mild, moderate, and vigorous physical activity in a typical week (Godin and Shepard, 1985). The scale has shown good test–retest reliability after 2 weeks (*r* = 0.74) and good convergent validity with maximum oxygen intake (*r* = 0.83) and values of body fat (*r* = 0.85; Godin and Shepard, 1985). For the analysis, the total number of minutes spent doing moderate and vigorous PA weekly were computed (Kushi et al., 2012).

#### 5-A-Day: Fruit and Vegetable Intake

The 5-A-Day measure is a one item questionnaire developed by the ACS to measure adherence to the recommended five servings of fruits and vegetables a day (Smith et al., 2007). Respondents indicated in a typical week in the past month, how many days per week they consumed the daily five servings of fruits and vegetables.

#### Self-Efficacy

The 8-item Perceived Health Competence Scale (Smith et al., 1995) has shown good internal consistency (*α* = 0.82–0.90) and construct validity in healthy and chronically ill samples (Smith et al., 1995). An example of an item is “*I’m generally able to accomplish my goals with respect to my health*.” Higher total scores indicate a greater self-efficacy to manage health.

#### Fear of Cancer Recurrence

Fear of cancer recurrence was assessed using the 9-item Fear of Cancer Recurrence Inventory-Short Form (FCRI-SF; Simard and Savard, 2009). An example of an item is “*I am worried or anxious about the possibility of cancer recurrence*.” The FCRI-SF ranges from 0 to 32, has good internal consistency (*α* = 0.89) and good test–retest reliability after 1 month (*r* = 0.80; Simard and Savard, 2009). The initial cut-off score for clinical FCR was 13 (Simard and Savard, 2009) but additional studies have suggested cut-off scores of 16 and 22 (Fardell et al., 2018).

### Data Analysis Strategy

Data were screened and cleaned using IBM SPSS 25. See Séguin Leclair et al. (2019) for detailed description of sample selection. Means, SD, and bivariate correlations were computed for all model variables. Statistical assumptions for regression analysis were verified.

Path analysis was conducted to test the hypothesized model (see Figure 1) using IBM AMOS at a level of significance *p* < 0.05. Path coefficients were standardized to facilitate comparison and interpretation of data. Bootstrapping with 2,000 samples and 95% CIs was used to calculate indirect effects. Model fit was established using the following goodness-of-fit indices with corresponding criteria: a small and non-significant chi-square likelihood ratio statistic (*χ*^2^), standardized root mean square residual (SRMR) ≤ 0.08, comparative fit index (CFI) ≥ 0.95, and root mean square error of approximation (RMSEA) ≤ 0.06 (Hayduk et al., 2007). Using the modification indices proposed by AMOS, additional regression weights were sequentially added to the model until the goodness-of-fit indices reached previously mentioned criteria. Concurrently, theoretical meaning was considered before the addition of parameters in the model. Adequate sample size was reached for the total sample (with 19 model parameters the minimum sample size required is *n* = 190), based on the suggested 10 participants/parameter (Kline, 2015). Given that variables met the normality assumption, the maximum likelihood estimation method was used.

## Results

### Study Sample

The 2,337 participants in this sample were mostly Caucasian (89.9%) women (60.4%) with college education or more (68%) and with a mean age of 56 at diagnosis. The most common cancer sites were breast (28.6%), prostate (21%), and colorectal (13.6%). See Table 1 for all participants’ socio-demographic and medical characteristics.

**Table 1:** Participant characteristics, *N* = 2,337.

Variable	*M*	*SD*
Age at diagnosis (years)	56.22	11.19
Time since diagnosis (years)	8.8	0.63
	*n*	*%*
Sex		
Male	936	39.6
Female	1,411	60.4
Ethnicity		
Caucasian	2,100	89.9
African American	116	5
Hispanic	66	2.8
Other	45	1.9
Not indicated/missing	10	0.4
Education		
High school or less	727	31.1
College or more	1,588	68
Not indicated/missing	22	0.9
Cancer type		
Breast	668	28.6
Prostate	490	21
Colorectal	317	13.6
Uterine	152	6.5
NHL	152	6.5
Melanoma	139	5.9
Kidney	127	5.4
Lung	103	4.4
Ovarian	100	4.3
Bladder	89	3.8
Cancer stage		
Stage 0–1	1,649	70.6
Stage 2–3	688	29.4
Civil status		
Married/cohabitating	1,813	77.6
Divorced/separated	226	9.7
Widowed	145	6.2
Single	145	6.2
Not indicated/missing	8	0.3
Household income		
0–9,999	57	2.4
10,000–19,999	144	6.2
20,000–39,999	456	19.5
40,000–74,999	749	32
75,000 or more	641	27.4
Not indicated/missing	290	12.4
Occupation		
Employed full-time	1,127	48.2
Employed part-time	196	8.4
Retired	577	24.7
Homemaker	148	6.3
Leave or unemployed due to illness	119	5,1
Unemployed	73	3.1
Student	9	0.4
Not indicated/missing	88	3.8

### Descriptive Statistics and Correlations for Model Variables

Table 2 displays the means, SDs, and bivariate correlations for the seven variables (illness consequences, control, timeline, physical activity, FVI, self-efficacy, and FCR) in the hypothesized model. Overall, survivors reported an average score of *M* = 11.4 (*SD* = 7.1) on the FCRI-SF, with 32.9% scoring above the clinical cut-off >13. The average number of days participants consumed five servings of fruits and vegetables per week was 3.31 days (*SD* = 2.17). Participants reported engaging in 97.59 (*SD* = 151.28) min of moderate to vigorous physical activity per week.

**Table 2:** Means, SDs, and bivariate correlations for the seven variables in the hypothesized model (*n* = 2,337).

Variable	*M*	*SD*	1	2	3	4	5	6	7
1. Consequences	48.47	11.06	-						
2. Control	13.53	4.48	−0.46^∗∗^	-					
3. Timeline	8.26	2.68	−0.20^∗∗^	0.17^∗∗^	-				
4. Physical activity	97.59	151.28	0.27^∗∗^	−0.32^∗∗^	−0.10^∗∗^	-			
5. Fruit and vegetable	3.31	2.17	0.12^∗∗^	−0.23^∗∗^	−0.06^∗∗^	0.16^*^	-		
6. Self-efficacy	3.57	0.63	0.48^∗∗^	−0.57^∗∗^	−0.28^∗∗^	0.32^**^	0.26^∗∗^	-	
7. FCR	11.40	7.08	−0.23^∗∗^	0.16	0.55^∗∗^	−0.09^**^	−0.05^∗^	−0.29^∗∗^	-

FCR, fear of cancer recurrence measured with the Fear of Cancer Recurrence Inventory-Short Form.

∗ *p* < 0.05;

∗∗ *p* < 0.01.

### Path Analysis

**Figure 2:**
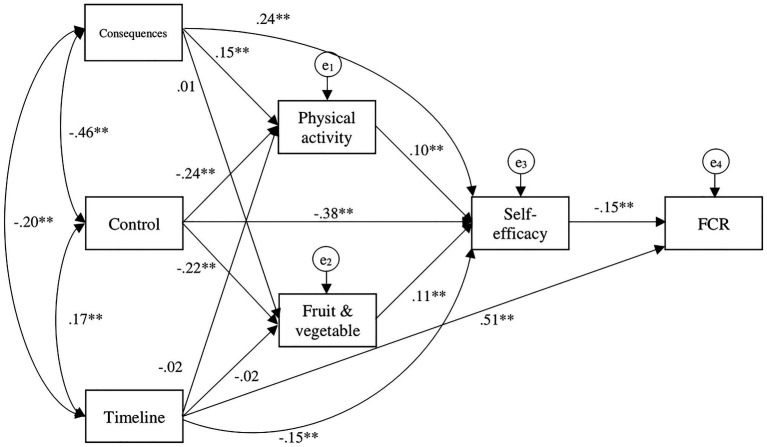
Final model path analysis diagram with standardized coefficients ^∗∗^*p* < 0.01.

The hypothesized model was tested but yielded poor goodness-of-fit indices, *χ*^2^ (9, *N* = 2,337) = 1718.72, *p* < 0.001; SRMR = 0.16; CFI = 0.49; RMSEA = 0.29. By adding four additional paths as suggested by the modification indices, the model fit improved [*χ*^2^ (5, *N* = 2,337) = 38.12, *p* < 0.001; SRMR = 0.02; CFI = 0.99; RMSEA = 0.05; see Figure 2]. The chi-square likelihood ratio statistic remained significant, but the model was deemed acceptable given its large sample size.

The final model indicated that both perceiving fewer illness consequences, and greater control over one’s health, were directly associated with higher PA (*β* = 0.15 and −0.24, *p* < 0.01, respectively) but also higher health self-efficacy (*β* = 0.24, −0.38, *p* < 0.01, respectively). Timeline was not directly associated with PA or FVI but was associated with lower health self-efficacy (*β* = −0.15, *p* < 0.01) and higher FCR (*β* = 0.51, *p* < 0.01). Both greater PA and FVI were directly associated with higher health self-efficacy (*β* = 0.10 and 0.11, *p* < 0.01, respectively), which in turn was directly associated with lower levels of FCR (*β* = −0.15, *p* < 0.01).

Small indirect effects were also observed between illness perception variables and health self-efficacy and FCR: timeline had an indirect effect on FCR (*β* = 0.06, *p* < 0.01), control had indirect effects on health self-efficacy (*β* = −0.01, *p* < 0.01) and FCR (*β* = 0.10, *p* < 0.01), and illness consequences also had indirect effects on health self-efficacy (*β* = 0.001, *p* < 0.01) and FCR (*β* = −0.02, *p* < −0.01). Last, PA and FVI had small indirect effects on FCR (*β* = −0.001 and −0.05, *p* < 0.01, respectively).

## Discussion

The goal of this study was to explore the relationship between health behaviors (PA and FVI) and FCR in a population-based sample of mixed long-term cancer survivors using the CSM and self-efficacy theory.

As hypothesized, survivors who perceived more control over health reported more PA and FVI. These results are congruent with the body of CSM literature, where perceived control is an important predictor of health behaviors in chronically ill samples (Richardson et al., 2016; Hagger et al., 2017). Contrary to our hypothesis, illness consequences and timeline were, for the most part, uncorrelated with health behaviors. Also as hypothesized, cancer survivors reporting more health behaviors endorsed greater health self-efficacy, which in turn correlated with lower levels of FCR.

The relationships between health behaviors and the CSM illness attributes lends tentative support for their conceptualization as problem-focused coping behaviors used to manage illness outcomes. While emotion-focused coping tends to be associated with consequences and timeline, problem-focused coping is strongly related to perceived control (Richardson et al., 2016; Hagger et al., 2017), which the present study also found.

Additional paths revealed another important factor predicting FCR. Specifically, timeline was the variable that showed the strongest association with FCR, congruent with previous studies (Phillips et al., 2013; Moon et al., 2017). In addition, survivors who perceived more illness consequences, their cancer to be chronic, and less health-related control reported lower self-efficacy, which in turn was related to higher FCR. Of course, no causality can be assumed in the present preliminary cross-sectional study and it is possible that survivors with lower FCR report greater health self-efficacy, which could contribute to their adherence to recommended health behaviors. Longitudinal and experimental studies are needed to confirm the present findings.

### Study Limitations

Although the American Cancer Society had developed a protocol to obtain an optimal sample of American cancer survivors, individuals who completed the questionnaire packages have specific characteristics, such as being female, White, and higher education (Smith et al., 2007). This limits the generalizability of findings. In addition to its cross-sectional design, this study captured FCR, CSM, self-efficacy, and health behaviors later in the cancer survivorship trajectory. While FCR severity was found to be stable across the three waves of data of the SCS-I (Séguin Leclair et al., 2019), factors in the CSM model have been shown to fluctuate over time (Leventhal et al., 2016). Furthermore, information of disease recurrence/progression was not available. Future studies should monitor health behaviors, including changes in these behaviors from pre-diagnosis, and CSM factors periodically throughout the survivorship trajectory, controlling for possible recurrence/progression (Leventhal et al., 2016). While the questionnaires used to assess illness representation components in this study were adequate measures of the constructs, the Revised Illness Perceptions Questionnaire was not used in this study (Moss-Morris et al., 2002). Given that this measure is commonly used in other CSM studies, this limits generalizability across studies. Moreover, the perceived cause of cancer recurrence, another component of illness representation in the CSM model, was not included in the original SCS-I survey questionnaire. As previous studies have consistently shown, causal attribution of cancer to poor diet or lack of exercise predicts adherence to health behaviors (Mullens et al., 2004; Costanzo et al., 2011; Burris et al., 2012); the absence of this measure might limit the understanding of factors predicting health behaviors. This study only focused on one possible emotional outcome outlined in CSM, FCR. Future studies could include additional outcomes that are associated with illness representation such as psychological distress (Llewellyn et al., 2007).

### Future Directions

It would be important to replicate the present study using a longitudinal design that would allow taking into account FCR trajectories, given the emerging empirical evidence that cancer survivors can be classified into three FCR severity sub-groups: low, moderate, and high, which have distinct survivorship profiles and patient characteristics (Simard and Savard, 2009; Simonelli et al., 2017; Séguin Leclair et al., 2019). As part of the larger study (Séguin Leclair et al., 2019), we found three stable FCR trajectories (low, moderate, and high); furthermore, cancer survivors in the high FCR trajectory group engaged in less health behaviors than other survivors. Therefore, it is possible that health behaviors may play less of a role in modulating FCR for those with persistently high FCR. It would also be interesting to see if these relations we observed in the present sample differ by sex, ethnicity, or cancer stage.

### Clinical Implications

The results of this study offer preliminary evidence that engaging in PA or consuming fruits and vegetables may increase health self-efficacy, which in turn may be beneficial to manage FCR in long-term survivors. In a previously published paper using the three waves of data from the SCS-I, we also found that survivors who quit smoking at T1 reported significant reductions in FCR at T3 compared to those who continued smoking (Westmaas et al., 2019). Together, these results support stepped care models that propose that all survivors would receive educational programs on health behaviors to help manage FCR (Stanton, 2012). The CSM showed a good fit across participants, further supporting its use in current FCR conceptualizations (Fardell et al., 2016; Simonelli et al., 2017) and interventions (Maheu et al., 2016; Butow et al., 2017). Timeline (i.e., perceiving cancer to be chronic) stood out as the strongest correlate of FCR in the model; hence interventions targeting the perceived chronicity of cancer may help cancer patients manage their FCR. Furthermore, improving cancer survivors’ self-efficacy to manage their health could be an FCR intervention target.

## Conclusion

Overall, the CSM, with the addition of self-efficacy, showed a good fit in the present sample of mixed long-term cancer survivors, in line with recent recommendations to combine both theoretical frameworks to improve the management of chronic illness (Breland et al., 2020). This study found that engaging in recommended health behaviors was correlated with increased health self-efficacy. Timeline and health self-efficacy were related to FCR and could be incorporated in future FCR interventions.

## Data Availability Statement

The raw data supporting the conclusions of this article will be made available by the authors, without undue reservation.

## Ethics Statement

The studies involving human participants were reviewed and approved by the Institutional Review Board of Emory University (Atlanta, GA, United States), for each state, including the Connecticut Department of Public Health Human Investigation Committee, and the University of Ottawa Research and Ethics Board (Ottawa, Ontario). The patients/participants provided their written informed consent to participate in this study.

## Author Contributions

All authors listed have made a substantial, direct and intellectual contribution to the work, and approved it for publication.

### Conflict of Interest

The authors declare that the research was conducted in the absence of any commercial or financial relationships that could be construed as a potential conflict of interest.
